# EEG–fNIRS-Based Emotion Recognition Using Graph Convolution and Capsule Attention Network

**DOI:** 10.3390/brainsci14080820

**Published:** 2024-08-16

**Authors:** Guijun Chen, Yue Liu, Xueying Zhang

**Affiliations:** College of Electronic Information and Optical Engineering, Taiyuan University of Technology, Taiyuan 030024, Chinatyzhangxy@163.com (X.Z.)

**Keywords:** electroencephalogram (EEG), functional near-infrared spectroscopy (fNIRS), graph convolution network, capsule attention network, emotion recognition

## Abstract

Electroencephalogram (EEG) and functional near-infrared spectroscopy (fNIRS) can objectively reflect a person’s emotional state and have been widely studied in emotion recognition. However, the effective feature fusion and discriminative feature learning from EEG–fNIRS data is challenging. In order to improve the accuracy of emotion recognition, a graph convolution and capsule attention network model (GCN-CA-CapsNet) is proposed. Firstly, EEG–fNIRS signals are collected from 50 subjects induced by emotional video clips. And then, the features of the EEG and fNIRS are extracted; the EEG–fNIRS features are fused to generate higher-quality primary capsules by graph convolution with the Pearson correlation adjacency matrix. Finally, the capsule attention module is introduced to assign different weights to the primary capsules, and higher-quality primary capsules are selected to generate better classification capsules in the dynamic routing mechanism. We validate the efficacy of the proposed method on our emotional EEG–fNIRS dataset with an ablation study. Extensive experiments demonstrate that the proposed GCN-CA-CapsNet method achieves a more satisfactory performance against the state-of-the-art methods, and the average accuracy can increase by 3–11%.

## 1. Introduction

As a subjective feeling, emotion cannot be defined as a unitary concept. Existing literature suggests that it is feasible to qualify, contextualize, and define functionally discrete emotions like happy, sad, fear, calm, etc. Specifically, many researchers affirm that emotion is a psychological state and affects people’s cognition, behavior, and even physiological response [1]. So, emotion plays an important role in interpersonal communication, and emotion recognition has broad application fields in daily life, such as human–computer interaction, education, entertainment, clinical diagnostics, etc. Traditional emotion recognition mainly uses facial expression, body posture, and voice intonation, but they are easily controlled by people’s subjective consciousness. When people’s inner feeling is inconsistent with their outward manifestation, the emotion recognition system will make the wrong judgment. However, physiological signals can objectively reflect people’s emotional state and are mainly controlled by the nervous system [2]. Specifically, the brain is the root of feeling emotions, and recent studies have found that emotions result from coordinated activity between the cerebral cortex and the subcortical nerve [3]. Due to being noninvasive and user-friendly, electroencephalogram (EEG) and functional near-infrared spectroscopy (fNIRS) are commonly used physiological signals related to neural activity in the brain [4]. EEG signals have high temporal resolution, and fNIRS signals have high spatial resolution, and both of them can reflect the emotional cognitive process of the brain [5]. The EEG records the electrical activities in the brain from electrodes placed on the scalp, which can monitor emotion-related brain dynamics directly. The fNIRS uses near-infrared light to measure the concentration change of oxygenated hemoglobin (HbO) and deoxygenated hemoglobin (HbR) indirectly during emotion processing, and it is not affected by the EEG.

There have been several works devoted to EEG/fNIRS-based emotion recognition from two aspects: feature extraction methods and recognition models [6,7]. The state-of-the-art methods for emotion recognition using EEG/fNIRS are listed in Table 1. For EEG data, Chen et al. [8] extracted six kinds of time and frequency domain features to classify different emotions using support vector machines (SVMs) and obtained the best accuracy in differential entropy and function connectivity features with 10-fold cross-validation (CV). To learn discriminative spatial–temporal emotional information, a multi-feature fusion convolution neural network (CNN) inspired by GoogleNet was proposed [9], which achieved average emotion recognition accuracies of 80.52% and 75.22% in the valence and arousal tasks of the DEAP dataset by leave-one-subject-out cross-validation (LOSO CV). Moreover, to learn the interaction of brain regions, Du et al. [10] proposed a multidimensional graph convolution network (MD-GCN), which integrates the temporal and spatial characteristics of EEGs and can classify emotions more accurately. Zhang et al. [11] proposed a multi-frequency band EEG graph feature extraction and fusion method for emotion recognition. They achieved average accuracies of 97.91%, 98.46%, and 98.15% for the arousal, valence, and arousal–valence classifications for the DEAP dataset. To effectively combine the spatial, spectral, and temporal information of an EEG, Gong et al. [12] proposed a novel attention-based convolutional transformer neural network (ACTNN) and achieved an average recognition accuracy of 98.47% and 91.90% on SEED and SEED-IV datasets. Wei et al. [13] proposed a Transformer Capsule Network (TC-Net), which mainly contained a Transformer module to extract EEG features and an Emotion Capsule module to refine the features and classify the emotion states. Liu et al. [14] proposed a multi-level, feature-guided capsule network (MLF-CapsNet) for multi-channel, EEG-based emotion recognition, and results showed that the proposed method exhibited higher accuracy than the state-of-the-art methods. For fNIRS data, Bandara et al. [15] demonstrated the capability of discriminating affective states on the valence and arousal dimensions and provided a higher F1-score for Valence. Hu et al. [16] reported 10 recognizable, typical kinds of positive emotions using fNIRS and provided support for implementing a more fine-grained positive emotion recognition. Si et al. [17] constructed a dual-branch joint network (DBJNet) to decode fNIRS-based emotion states, which can effectively distinguish positive versus neutral versus negative emotions (accuracy is 74.8%; F1 score is 72.9%).

On the other hand, hybrid EEG–fNIRS was proposed to further enhance the performance of emotion recognition. According to [18], EEG activity was intrinsically associated with the cortical hemodynamic (fNIRS) responsiveness to negative emotional patterns. Rahman et al. [19] discussed the applicability of EEG and fNIRS biomarkers for the assessment of emotional valence. Chen et al. [20] exploited the temporal convolutional network (TC-ResNet) to recognize the emotional features of EEG–fNIRS, which showed significant accuracy gains of 0.24% for the EEG and 8.37% for fNIRS. Sun et al. [21] proposed a method to jointly evaluate fNIRS and EEG signals for affective state detection. They showed that the proposed hybrid method outperforms fNIRS-only and EEG-only approaches. The hybrid EEG–fNIRS is considered to combine the advantages of the two modalities and compensate for the limitations of each modality; however, the possibilities of EEG–fNIRS feature fusion to discriminate the specific emotion states have not been investigated comprehensively.

**Table 1:** State-of-the-art methods for emotion recognition using EEG–fNIRS.

Method	Data	Features	Protocol	Accuracy (%)
SVM [8]	EEG from DEAP	time and frequency domain features	10-fold CV	89.94
CNN [9]	EEG from DEAP	time and frequency domain features	LOSO CV	80.52 valence, 75.22 arousal
MD-GCN [10]	EEG from SEED/SEED-IV	temporal and spatial features	LOSO CV	92.15/90.83
GC-F-GCN [11]	EEG from DEAP	differential entropy	5-fold CV	98.46 valence, 97.91 arousal
ACTNN [12]	EEG from SEED/SEED-IV	differential entropy	10-fold CV	98.47/91.90
TC-Net [13]	EEG from DEAP	continuous wavelet transform	10-fold CV	98.76 valence, 98.81 arousal
MLF-CapsNet [14]	EEG from DEAP	preprocessed EEG	10-fold CV	97.97 valence, 98.31 arousal
SVM [15]	fNIRS	slope, min, max, etc. from HbO and HbR	4-fold CV	-
SVM [16]	fNIRS	HbO/HbR	6-fold CV	83.69/79.06
DBJNet [17]	fNIRS	HbO	LOSO CV	74.80
TC-ResNet [20]	EEG, fNIRS	spectral features from EEG-HbO	-	99.81
SVM [21]	EEG, fNIRS	PSD from EEG, mean, median, etc. from HbT, HbO, HbR	leave-one-out CV	80.00

At the present, emotion recognition based on deep learning has achieved great progress, especially emotion feature learning and fusion domains. To comprehensively examine the efficiency of hybrid EEG–fNIRS, various deep learning algorithms have been developed to analyze patterns from EEG and fNIRS data. However, there are still two problems with EEG–fNIRS feature fusion for emotion recognition using a deep learning algorithm. One main issue is requiring massive data to train the deep learning model. If the number of samples is insufficient, the model will be affected by overfitting, the training difficulty will increase, and the recognition accuracy will decrease. Another challenge is that the input and output neurons of the traditional CNN are in scalar form, which fails to retain the valuable spatial relationship between features and affects the recognition accuracy. The input and output neurons of the capsule network are vectors, which can retain the exact spatial position information. To overcome the information loss, it abandons the pooling layer structure and only needs a few data to train a relatively good network. Thus, a graph convolution and capsule attentional network (GCN-CA-CapsNet) model was proposed in this study. Firstly, the EEG and fNIRS features were fused by graph convolution. And then, the capsule attention module was used to assign different weights to the capsule network, and EEG–fNIRS features from different depths were integrated efficiently.

The main contributions of this paper are given as follows.

(1) A novel EEG–fNIRS-based emotion recognition framework using a graph convolution and capsule attention network, namely GCN-CA-CapsNet, is introduced;
(2) The CNN layer is replaced with the GCN layer, which can extract and fuse the graph structure features and spatial correlation from the EEG and fNIRS;
(3) The capsule attention mechanism is proposed to give different primary capsules to different attention weights, and the primary capsules with higher quality are selected to generate better classification capsules.

The rest of the paper is organized as follows: The materials and methods are demonstrated in Section 2. Then, the experimental results and analysis are presented in Section 3. Finally, the conclusion and discussion are provided in Section 4 and Section 5, respectively.

## 2. Materials and Methods

### 2.1. EEG–fNIRS Dataset and Preprocessing

**Figure 1:**
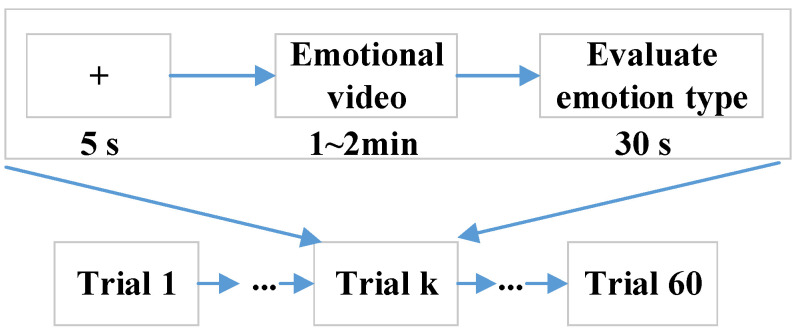
Experimental paradigm.

Fifty college students, including 25 males (age: 22.92 ± 1.71) and 25 females (age: 24.12 ± 1.67), volunteered for this study. All participants self-reported normal or corrected-to-normal vision and normal hearing in the experiments. Each participant gave written informed consent prior to participation, and all of them self-identified as right-handed and self-reported to have no history of mental illnesses or drugs, which were the inclusion criteria. The EEG–fNIRS data from all participants were included in the data analysis. Each participant was asked to sit in a comfortable chair facing a computer screen and assigned to watch 60 emotional video clips, including sad, happy, calm, and fear, and each kind of emotion had 15 video clips. The selected video clips were pre-assessed from the dimensions of arousal and valence according to Self-Assessment Manikin (SAM) scores (1–9) from 20 subjects, who were not the experimental subjects. The detailed data description can be obtained from https://gitee.com/tycgj/enter. The experimental protocol is shown in Figure 1. Each video clip lasted 1~2 min, and then the participants evaluated the type of emotion within 30 s by filling out an evaluation form. Each participant was instructed in the experimental procedure in detail before performing the experiment.

**Figure 2:**
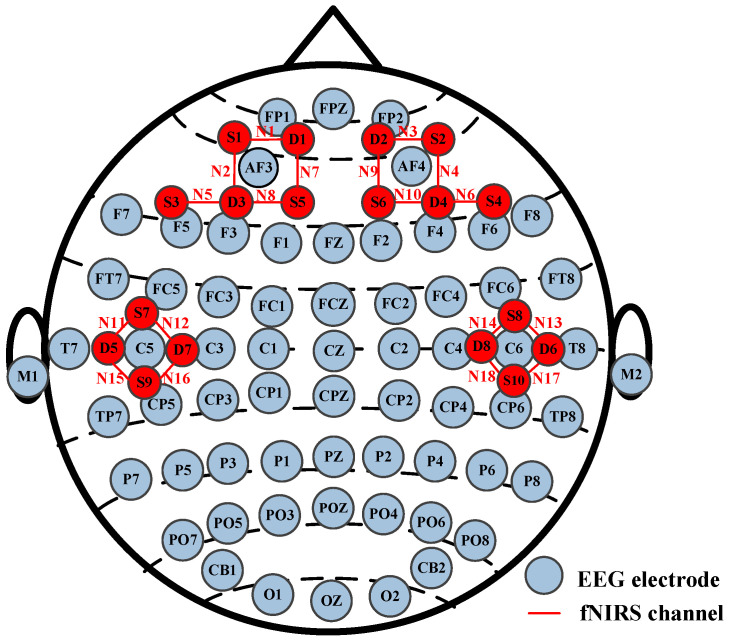
EEG and fNIRS channel distribution map.

Both EEG and fNIRS sensors were placed on each participant’s scalp, and there was no contact between these two hardware devices (Neuroscan SynAmps2 from Neuroscan USA, Ltd. Charlotte, NC, USA and NirSmart from DanYang HuiChuang Medical Equipment Co., Ltd., Danyang, China). The EEG was recorded with a 1000 Hz sampling rate and 64 channels, and the fNIRS with an 11 Hz sampling rate and 18 channels. The EEG and fNIRS channel distribution map is shown in Figure 2. During the experiment, each participant was required to minimize head movements to avoid signal artifacts. The study was conducted in accordance with the Declaration of Helsinki and approved by the Institutional Review Board of Taiyuan University of Technology. After the experiment, each participant was paid a certain amount of money in the study.

Furtherly, we used the EEGLAB v13.4.4b toolbox in MATLAB R2019b to preprocess the raw EEG data as follows. The EEG data were converted to a bilateral mastoid reference and bandpass filtered at 0.5–45 Hz cutoff frequency using a Hamming windowed sinc FIR filter. The continuous EEG data were divided into epoch data by extracting an emotion event, and 2 s baseline correction was applied. And then, the epoch data were segmented into multiple samples with a window length of 3 s and a step length of 1.5 s. Finally, an independent component analysis was run, and specified electrooculogram (EOG) artifact components were removed from the EEG data [22].

Similar to the EEG, the raw fNIRS data were corrected using 2 s baseline and bandpass filtered at 0.01–0.2 Hz cutoff frequency. The artifacts’ segments of sharp change were identified and removed. And then, for the hemodynamics analysis, we converted the optical density to the relative concentration changes of oxygenated hemoglobin (HbO) and deoxygenated hemoglobin (HbR) with the modified Beer–Lambert Law [23]. Due to the more reliable measurement of cerebral blood response by HbO than HbR, the HbO-based classifications in general showed better performance than the HbR-based ones [16]. So, the HbO data were used and segmented into multiple samples in the same way as the EEG signals in this paper.

### 2.2. Feature Extraction

After preprocessing the EEG data, we extracted differential entropy (DE) features of each sample in five frequency bands, including δ (0.5–4 Hz), θ (4–8 Hz), α (8–13 Hz), β (13–30 Hz), and γ (30–45 Hz). The DE feature was mostly used in EEG-based emotion recognition and illustrated to be effective. The DE can measure the complexity of signals, which can be expressed as follows:

(1) $$DE\left(x\right)=-\int_{-\infty }^{\infty }\frac{1}{\sqrt{2\pi \sigma^{2}}}e^{-\frac{\left(x-\mu^{2}\right)}{2\sigma^{2}}}\log\left(\frac{1}{\sqrt{2\pi \sigma^{2}}}e^{-\frac{\left(x-\mu^{2}\right)}{2\sigma^{2}}}\right)dx=\frac{1}{2}\log\left(2\pi e\sigma^{2}\right)$$

where the EEG sample x should satisfy the Gaussian distribution with $N\left(\mu ,\sigma^{2}\right)$. It has been proven that the sub-band signals can meet the Gaussian distribution hypothesis [24].

Because the fNIRS signal mainly reflects the hemodynamic characteristics in time series, for the HbO sample y(i),i=1⋯N, *N* is the sample length. We extracted five kinds of features from different channels, i.e., mean $\bar{y}$, variance $\sigma^{2}$, skewness s, power spectrum density (PSD), and differential entropy (DE). These features are defined as follows:

(2) $$\bar{y}=\frac{1}{N}\sum_{i=1}^{N}\left(y(i)\right)$$

(3) $$\sigma^{2}\left(y\right)=\frac{1}{N}\sum_{i=1}^{N}{\left(y(i)-\bar{y}\right)}^{2}$$

(4) $$s\left(y\right)=\frac{1}{N\sigma^{3}}\sum_{i=1}^{N}{\left(y(i)-\bar{y}\right)}^{3}$$

(5) $$PSD\left(y\right)=\frac{1}{N}{\left|FFT\left(y(i)\right)\right|}^{2}$$

(6) $$DE\left(y\right)=\frac{1}{2}\log\left(2\pi e\sigma {\left(y\right)}^{2}\right)$$

where FFT represents the Fast Fourier Transform operation.

### 2.3. GCN-CA-CapsNet Model

**Figure 3:**
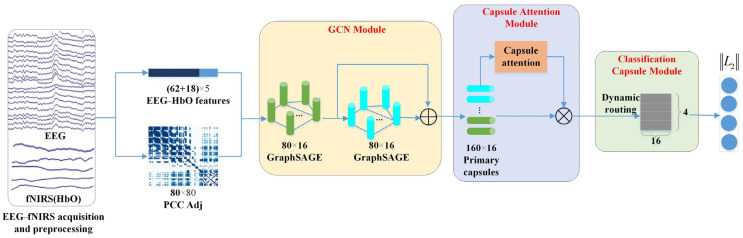
Schematic of the proposed GCN-CA-CapsNet framework.

On the whole, our proposed graph convolution and capsule attentional network (GCN-CA-CapsNet) model can be divided into three crucial components: the GCN module, capsule attention module, and dynamic routing-based classification capsule module. Figure 3 illustrates the framework of the proposed GCN-CA-CapsNet for EEG–fNIRS-based emotion recognition, which will be introduced in detail as follows. The code related to this model is available at https://gitee.com/tycgj/gcn-ca-capsnet.

#### 2.3.1. GCN Module

The graph convolutional network (GCN) can be used to process graph data by combining convolution with graph theory, which provides an effective way to explore the spatial relationships among multiple EEG–fNIRS channels. In this module, two-layer GCNs are carried on the EEG and HbO features extracted in Section 2.2 and generate high-level EEG–fNIRS representations.

A graph can be defined as $G=\left\{V,E\right\}$, in which V represents the set of nodes with the number *C*, and E represents the set of edges connecting these nodes. In this study, the EEG–fNIRS feature matrix $X\in R^{C\times d}$ is used as the set of nodes, where *C* = 80 is the channel number, and *d* = 5 is the feature dimension. Let $Adj\in R^{C\times C}$ denote an adjacency matrix describing the connection relationships between any two nodes in V. Commonly, the connection relationships can be characterized by the Pearson correlation coefficient (PCC), phase locking value (PLV), Granger causality (GC), etc. Compared with the PLV and GC, the PCC is used as an adjacency matrix with higher classification accuracy, referring to the experimental results in Section 3.1, so we employ the PCC to construct the adjacency matrix among the EEG–fNIRS channels.

The PCC measures the similarity of two vectors and its value is between −1 and 1. The positive or negative value of the PCC can indicate whether the relationship between two vectors is a positive or negative correlation. As the absolute value of the PCC increases, the correlation between the two variables becomes stronger. If the PCC is 0, it indicates that there is no correlation between the two vectors. Assuming that any two channels of EEG–fNIRS signals $x_{i},x_{j}$, and then we can define

(7) $$PCC_{x_{i},x_{j}}=\frac{\mathrm{cov}\left(x_{i},x_{j}\right)}{\sigma_{x_{i}}\sigma_{x_{j}}}=\frac{E\left(\left(x_{i}-\mu_{x_{i}}\right)\left(x_{j}-\mu_{x_{j}}\right)\right)}{\sigma_{x_{i}}\sigma_{x_{j}}}$$

where cov represents the covariance, *E* represents expectation, and σ represents the standard deviation. Since the sampling rate between the EEG and fNIRS is different; the EEG signals are down sampled to 11 Hz during the calculation of the PCC, which is consistent with the sampling rate of the fNIRS signals.

It is notable that the used GCN structure is GraphSAGE [25]. Briefly, there are two main operations in this network: the sampling and aggregation of neighbor nodes. For a given node, relevant neighbor node features are selected according to the adjacency matrix *A*, and then a new node feature is obtained through an aggregation operation. The new node feature not only includes the information of previous nodes, but also aggregates the features of neighboring nodes. In the process of generating new nodes, the node features of both the EEG and fNIRS are aggregated, and the feature fusion of the EEG and fNIRS is realized. Compared with traditional graph convolution, GraphSAGE can extract the local features of graph data. For the GraphSAGE with mean aggregation, the formula of the node update can be expressed as follows:

(8) $$X_{i}^{k+1}=f\left(W\cdot MEAN\left(\left\{X_{i}^{k}\right\}\cup \left\{X_{j}^{k},\forall j\in N\left(i\right)\right\}\right)\right)$$

where $X_{i}^{k+1}$ represents the feature of node *i* in layer *k* + 1, $X_{i}^{k}$ represents the feature of node *i* in layer *k*, $X_{j}^{k},\forall j\in N\left(i\right)$ represents the feature vector of node *j* relevant with node *i* in layer *k*, *W* represents the parameter matrix, and *f* represents the nonlinear activation function.

In the first GCN layer, the input dimension is 5, the output dimension is 16, and the number of nodes is 80 (i.e., 62 EEG channels and 18 fNIRS channels). The node features from the EEG and fNIRS are aggregated to generate new node features according to the adjacency matrix. In the second GCN layer, the features from the first GCN layer are aggregated, and the output dimension is 16; the number of nodes is 80. Then, the GCN module eventually generates 160 16D features by concatenating two layers of output features, which are used to construct the primary capsules.

#### 2.3.2. Capsule Attention Module

**Figure 4:**
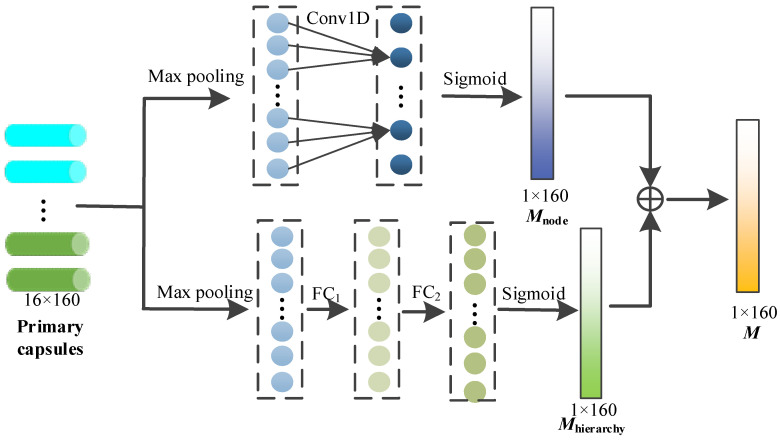
Capsule attention mechanism.

In this module, through the capsule attention mechanism, the primary capsules containing different depth node features are assigned different weights, so that we can pay more attention to the important primary capsules, and the specific process is shown in Figure 4.

The set of primary capsules can be represented as follows, and *k* = 160.

(9) $$U=\left\{u_{1},u_{2},\cdots ,u_{k}\right\}$$

To generate the capsule attention weight, each capsule is first maximized by pooling layer, and the maximum of each capsule is obtained.

(10) $$max_{k}=\mathrm{maxplooing}(u_{k})$$

The above maximums are concatenated as follows:

(11) $$U^{\prime }=contact(max_{1},max_{2},\cdots ,max_{k})$$

After that, the above information is exchanged across nodes through 1D convolution with a kernel size of 3, and the sigmoid function is used to provide nonlinear activation. The node attention weight is calculated via

(12) $$M_{node}=\mathrm{sigmoid}(conv1D(U^{\prime }))$$

On the other hand, the capsule attention needs to be designed to generate the weights to the primary capsules from different hierarchies (i.e., primary capsules from different GCN layers). The primary capsules consist of node features at different depths, and these features play different roles in the subsequent dynamic routing mechanism. After the max pooling of each capsule, the hierarchy weight *M_hierarchy_* is calculated through the two fully connected layers and sigmoid activation function as follows.

(13) $$M_{hierarchy}=\mathrm{sigmoid}(FC_{2}\left({FC}_{1}\left(U^{\prime }\right)\right)$$

The capsule attention weight *M* is finally obtained by adding the node weight and the hierarchy weight, and the size is 1 × 160.

(14) $$M=M_{node}+M_{hierarchy}$$

Finally, the capsule attention weight *M* is multiplied with the primary capsule as the output of the capsule attention module.

(15) $$U^{\prime \prime }=U⊗M$$

#### 2.3.3. Dynamic Routing-Based Classification Capsule Module

For the input and output vector of a capsule, the lower-level capsule is connected to the higher-level capsule by a dynamic routing algorithm. In the capsule attention module, we get 160 16D primary capsules as the input of the dynamic routing. To classify four kinds of emotions, the output of the dynamic routing is 4 16D classification capsules. The outputs of four classification capsules, calculated through the L2 norm and then activated by Softmax, represents the probability of emotion category, and is used in the calculation of the subsequent loss function.

**Figure 5:**
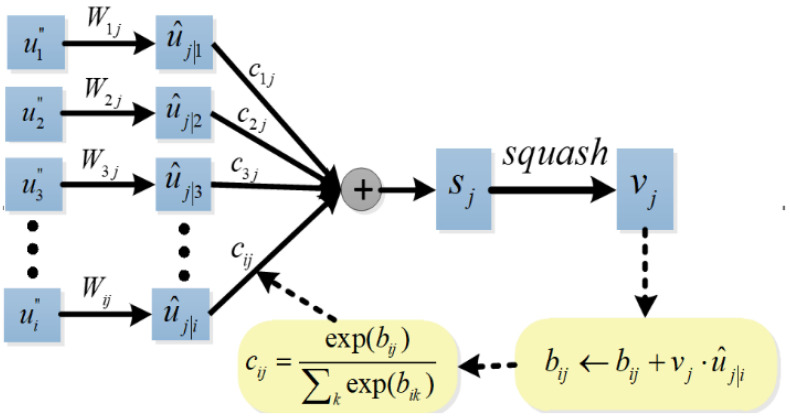
Dynamic routing algorithm.

The dynamic routing algorithm is shown in Figure 5. Firstly, the prediction vector ${\hat{u}}_{j|i}$ is produced by multiplying the output $u_{i}^{\prime \prime }$ of the capsule attention module with a weight matrix $W_{ij}$, $i\in \left\{1,2,\cdots ,160\right\}$, $j\in \left\{1,2,\cdots ,4\right\}$.

(16) $${\hat{u}}_{j|i}=W_{ij}u_{i}^{\prime \prime }$$

And then, a weighted sum over all prediction vectors is calculated

(17) $$s_{j}=\sum_{i}c_{ij}{\hat{u}}_{j|i}$$

where $c_{ij}$ are coupling coefficients between capsule *i* and capsule *j* in the higher layer that are determined by the iterative dynamic routing process. The coupling coefficients sum to 1 and are determined by the routing softmax

(18) $$c_{ij}=\frac{\exp(b_{ij})}{\sum_{k}\exp(b_{ik})}$$

where *b_ij_* are the log prior probabilities that capsule *i* should be coupled to capsule *j*, and *b_ij_* are updated iteratively according to Equation (20). The initial values of *b_ij_* are 0.

Finally, we use a non-linear squashing function to ensure the length of the output vector from a classification capsule between 0 and 1, and it can be used to represent the classification probability.

(19) $$v_{j}=\frac{{\left\|s_{j}\right\|}^{2}}{1+{\left\|s_{j}\right\|}^{2}}\frac{s_{j}}{\left\|s_{j}\right\|}$$

The output *v_j_* is multiplied with the prediction vector ${\hat{u}}_{j|i}$ to get a scalar, which is used to update the *b_ij_* until the set number of iterations is reached. Sabour et al. suggested that better convergence can be obtained by using three routing iterations than one iteration [26]. Therefore, we set the maximum number of routing iterations as 3.

(20) $$b_{ij}\leftarrow b_{ij}+{\hat{u}}_{j|i}\cdot v_{j}$$

When the predictions of the lower capsule and the higher capsule are consistent, the values of the *b_ij_* and *c_ij_* become larger, and when the predictions are inconsistent, the value of the *c_ij_* becomes smaller. Thus, the lower capsule can send its information to the classification capsule that is consistent with its predictions by adjusting the coupling coefficient.

The margin loss function is used to optimize the proposed GCN-CA-CapsNet model, which is defined as follows:

(21) $$L_{j}=T_{j}\max{\left(0,m^{+}-\left\|v_{j}\right\|\right)}^{2}+\lambda \left(1-T_{j}\right)\max{\left(0,\left\|v_{j}\right\|-m^{-}\right)}^{2}$$

where *T_j_* represents the target label, and *T_j_* = 1 if the label of the input sample is *j*; otherwise, *T_j_* = 0, $\left\|v_{j}\right\|$ represents the length of the classification capsule, and *m^+^* = 0.9 and *m^-^*= 0.1 represent the maximum and minimum margin thresholds, *λ* = 0.5.

## 3. Experimental Results and Analysis

### 3.1. Experimental Settings and Evaluation

All experiments are performed on the Pytorch 1.10.2 deep learning framework through NIVIDA Telsa T4 GPU. The environment platform includes Python 3.7 and CUDA 10.0. The model is trained by an Adam optimizer with a learning rate of 0.0001. The batch size of the training set is 32, and the maximum epoch is initiated to 200. In order to obtain reliable evaluation results, we perform a 5-fold cross-validation (CV) on the dataset from each subject. In each fold of the CV, 80% of samples are used to train the classifier, while the remaining samples are used for validating the results. To comprehensively evaluate the classification performance, the average accuracy (*Acc*) and standard deviation (*Std*) from the 5-fold calculations are used as evaluation metrics in the experiments, where $Acc=n_{correct}/n_{total}$, $n_{correct}$ is the number of correctly classified samples, and $n_{total}$ is the total number of samples. Meanwhile, the calculation amount (number of model parameters) and running time of the model are compared and analyzed.

**Figure 6:**
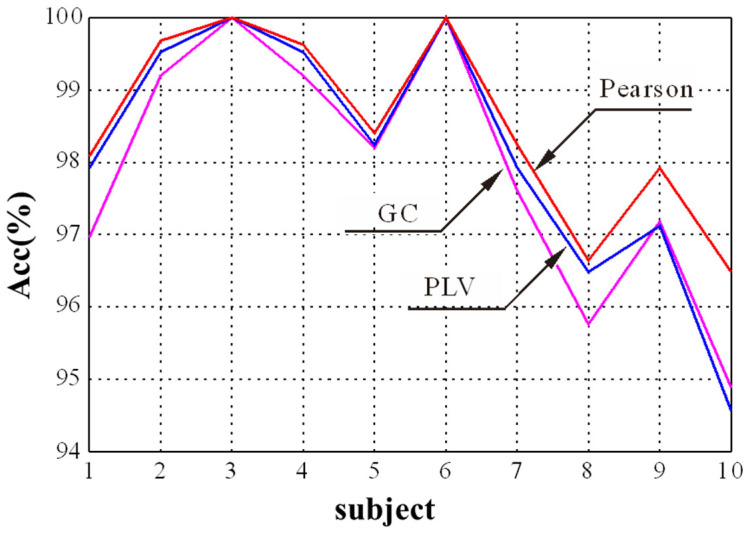
The recognition results of three adjacency matrices for different subjects.

In the GCN module, the selection of the adjacency matrix is crucial to find appropriate nodes to generate new node features through aggregation. The Pearson correlation, PLV, and GC were selected to explore the influence of adjacency matrices on the final emotion recognition performance. The experimental result for different adjacency matrices is shown in Figure 6. It shows that the proposed method obtains a better performance for the first 10 subjects when the Pearson correlation is used as the adjacency matrix compared with PLV and GC. It is consistent with the results in [27], which used the Pearson correlation coefficient as the adjacency matrix of graph convolutional network and achieved the best performance in EEG emotion recognition. Thus, we chose the Pearson correlation as the adjacency matrix in this study.

**Figure 7:**
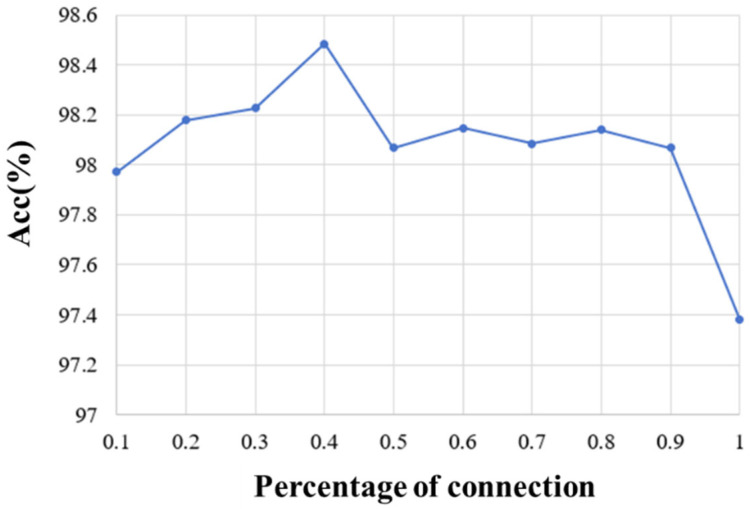
The recognition results of different percentages of Pearson correlation connection.

**Figure 8:**
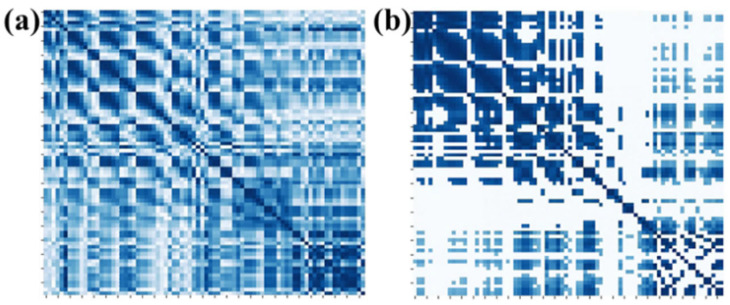
Adjacency matrices for (**a**) all connections and (**b**) 40% of connections.

On the other hand, we explored the selection of 10%, 20%, 30%, 40%, 50%, 60%, 70%, 80%, 90%, and 100% of full connection in adjacency matrix and analyzed the influence of different percentages of connection on the emotion recognition performance. The average accuracy of the first 10 subjects for different percentages of connection is shown in Figure 7. It can be seen that the proposed method obtains the best performance when 40% of the connections are selected. By removing part of the connection and selecting the most relevant connections, the adjacency matrix of this paper can be divided into three parts. The first part is the correlation between the EEG and EEG, the second part is the correlation between fNIRS and fNIRS, and the third part describes the correlation between the EEG and fNIRS. Therefore, the top 40% of connections with strong correlation in each part are selected as the adjacency matrix of our method. Figure 8a shows the adjacency matrix with all connections, and Figure 8b shows the adjacency matrix with the top 40% of connections.

### 3.2. Ablation Study

To further validate the effectiveness of each component of our GCN-CA-CapsNet framework, we perform an ablation study. The CapsNet is regarded as the baseline framework, and we superimpose the GCN and CA on the baseline framework in order, which aims to present their positive effect on emotion recognition. The competing frameworks are as follows.

(1) CapsNet: The CapsNet method includes two-layer CNN, a primary capsule module, and a classification capsule module;
(2) GCN-CapsNet: Compared with the CapsNet, this network replaces the CNN with the GCN, which is utilized to extract graph structure features from the EEG and fNIRS;
(3) GCN-CA-CapsNet: This network introduces the capsule attention mechanism, which gives different primary capsules to different attention weights for feature fusion.

**Figure 9:**
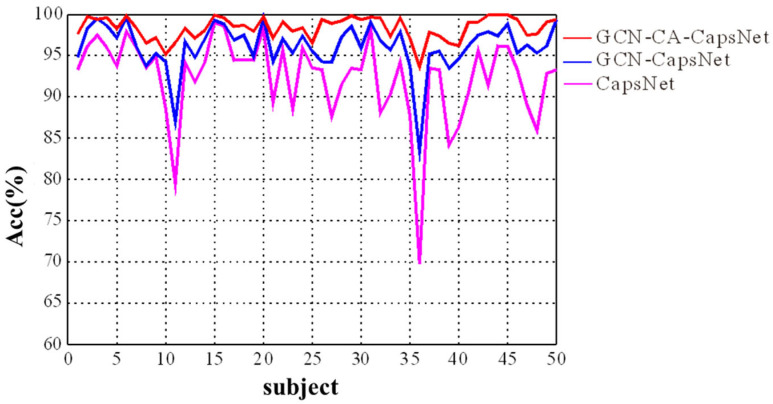
The recognition results of ablation experiment for different subjects.

We present the experimental results in Figure 9 and Table 2, which demonstrate the efficacy of each component of our GCN-CA-CapsNet model. As shown in Figure 9, the recognition accuracy of our GCN-CA-CapsNet model is significantly higher than the GCN-CapsNet model and CapsNet model for each subject (Paired samples *t*-test: *p* < 0.01). As can be seen in Table 2, compared with the CapsNet model, the average accuracy of the GCN-CapsNet model increases by 4.56%, and the standard deviation decreases by 2.41%. The recognition accuracy of sad, happy, calm, and fear are increased by 5.34%, 3.78%, 4.57%, and 4.58%, respectively. Meanwhile, the number of parameters and the running time of the GCN-CapsNet model are greatly reduced. This is mainly due to the fact that the GCN layer can reduce the invalid background information of the 2D EEG–fNIRS feature-mapping matrix used in the CNN layer of the CapsNet model. In other words, the GCN layer converts EEG–fNIRS features to high-level features that are then effectively used as inputs to the primary capsules. Furthermore, because the capsule attention mechanism is added, the number of parameters and the running time of the GCN-CA-CapsNet model increase by 31,314 and 38 s compared with the GCN-CapsNet model, respectively. But the average accuracy of GCN-CA-CapsNet reaches 97.91%, demonstrating the benefit of capsule attention weights. And compared with the CapsNet model, the number of parameters and running time of the GCN-CA-CapsNet model still decrease.

**Figure 10:**
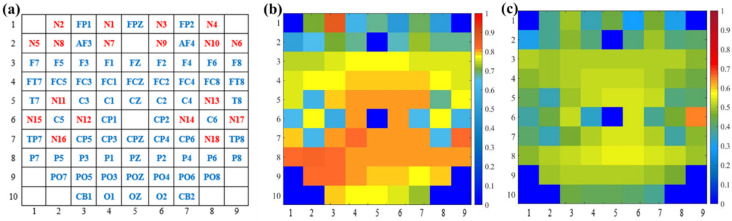
Visualization of capsule attention weights. (**a**) Capsule location mapping based on EEG–fNIRS channel distribution, (**b**) attention weights of capsules from first-layer GCN, and (**c**) attention weights of capsules from second-layer GCN.

To intuitively present the interpretability of the capsule attention weights, we apply the capsule location mapping, as shown in Figure 10a. Figure 10b shows the attention weights of capsules from the first layer of GCN, and Figure 10c shows the attention weights of capsules from the second layer of GCN based on Figure 10a. The weights are in one-to-one correspondence with the channel distribution of the EEG and fNIRS. It can be shown that more attention is paid to the primary capsule generated by the first GCN layer. The attention weights of capsules from the first GCN layer are about 0.8, and the attention weights of capsules from the second GCN layer are about 0.5. Moreover, the parietal and occipital lobes of the brain play a large role in emotion recognition, and the primary capsules generated by the EEG channels have higher weights than the primary capsules generated by the fNIRS channels. Through the capsule attention mechanism, the length of the primary capsule is changed. In the dynamic routing mechanism, the length of the capsule represents the category probability, and the primary capsule with higher quality helps to generate better classification capsules and improve the recognition performance.

### 3.3. Performance Comparison with Single EEG and Single fNIRS

To validate the efficacy of EEG–fNIRS feature fusion, we also applied the single modality of either EEG or fNIRS features for emotion recognition. As shown in Table 3, the average recognition accuracy of our GCN-CA-CapsNet method jointly using EEG–fNIRS features outperforms the accuracy using only one of them (96.17 for EEG and 84.66 for fNIRS). Compared with single EEG, the recognition accuracies of sad, happy, calm, and fear jointly using EEG–fNIRS features are significantly increased by 1.45%, 1.72%, 2.03%, and 1.74%, respectively (Paired samples *t*-test: *p* < 0.01). Moreover, compared with single fNIRS, the recognition accuracies of sad, happy, calm, and fear jointly using EEG–fNIRS features are significantly increased by 14.66%, 10.00%, 15.65%, and 12.67%, respectively (Paired samples *t*-test: *p* < 0.01). These results are consistent with the study in [21]. Our GCN-CA-CapsNet method could effectively fuse EEG–fNIRS features, which provide comprehensive information to boost emotion recognition performance. Since the concentration changes of HbO based on fNIRS are slow-varying signals, their ability to characterize emotional responses is limited.

**Figure 11:**
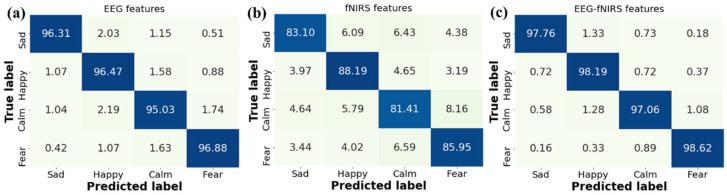
Confusion matrices for emotion recognition with different features: (**a**) EEG features, (**b**) fNIRS features, and (**c**) EEG–fNIRS features.

Further, we analyze the confusion matrices for emotion recognition with different features in Figure 11. It is observed that our GCN-CA-CapsNet method has high recognition accuracies on the happy and fear emotions for three kinds of features. This is due to the fact that both happy and sad video stimuli are more likely to evoke distinguishable emotional responses. The recognition accuracy of the four emotions increases when using the EEG–fNIRS features compared with single EEG features and single fNIRS features. The recognition accuracy of each emotion using fNIRS features is low because the fNIRS signal has only 18 channels, and the sampling frequency is 11 Hz; the emotion-related information is less, which is a limitation in this work. Our future work will try to increase the fNIRS channels and improve the accuracy.

### 3.4. Comparison with the State-of-the-Art Methods

Table 4 presents a performance comparison between the proposed GCN-CA-CapsNet method and recent deep learning methods, including GCN, TC-Net, MLF-CapsNet, and ST-CapsNet on our EEG–fNIRS emotion dataset. Five-fold cross-validation tests were performed in the same environment platform for all methods. The parameters of different methods are set based on the corresponding literatures. The GCN method uses two-layer GraphSAGE to learn EEG–fNIRS graph features. The TC-Net [13] contains a Transformer module to extract features and an Emotion Capsule module to refine the features and classify the emotion states. The MLF-CapsNet [14] incorporates multi-level feature maps in forming the primary capsules to enhance feature representation and uses a bottleneck layer to reduce the number of parameters. The ST-CapsNet [28] uses a capsule network with both spatial and temporal attention modules to learn discriminative features. In Table 4, the results show that our GCN-CA-CapsNet method illustrates the best recognition accuracy among the listed methods, which is 7.75%, 11.07%, 3.26%, and 3.90% higher than the other four comparison methods, respectively. The outstanding performance demonstrates that it is beneficial to combine graph convolution and capsule networks to fuse EEG–fNIRS features, especially the spatial correlation information, and design the capsule attention mechanism module to generate higher-quality primary capsules.

## 4. Discussion

Emotion is a complex coordinated activity between the cerebral cortex and the subcortical nerve; however, the mechanisms of how the brain processes emotions are still unclear. Currently, the neural basis of emotions is usually studied by using physiological signals [29,30]. Specifically, with the development of wearable devices, there is growing interest in combining noninvasive EEGs and fNIRS to gain a more thorough knowledge of the brain correlates of emotion [31]. The EEG can capture the rapid temporal dynamics of emotional response, and fNIRS can identify the more locally concentrated brain activity. The integration of EEG and fNIRS data offers a spatial–temporal complementarity for emotion recognition. In this paper, the accuracy of our GCN-CA-CapsNet method jointly using EEG–fNIRS features outperforms the accuracy of using only one of them. However, there are still three limitations. First, the inconsonant sampling frequency between the EEG and fNIRS leads to the difficulty of temporal feature fusion. The temporal alignment and data enhancement of fNIRS need to be considered when EEG–fNIRS features are fused [32,33]. Second, most hybrid EEG–fNIRS studies have focused on local brain regions [34]; the discrepancies in the recording locations between the EEG and fNIRS lead to the difficulty of spatial feature fusion. In this study, the fNIRS signal has only 18 channels involved in the prefrontal and temporal lobes, and the emotion-related spatial information is less than that of the EEG. Our future work will try to increase the fNIRS channels and improve the accuracy. Third, fNIRS signals can be converted to concentration changes of HbO and HbR. Both of them can characterize physiological responses to emotions [15,21]. In [16], the HbO-based classifications showed better performance than the HbR-based ones. Moreover, several previous studies [35,36] showed that the signal/noise ratio of HbO was much higher than that of HbR. To reduce the calculated amount, this study focused on the HbO concentration changes. In future work, we will further explore the influence of HbO–HbR on emotion recognition.

On the other hand, the multi-channel EEG–fNIRS signal is non-Euclidean data; traditional CNNs have limitations in extracting spatial connection features from EEG–fNIRS data [37]. In this paper, we address the challenges based on the GCN, attention mechanisms, and CapsNet [38]. The EEG–fNIRS data can be converted to a graph and then be processed to learn high-level emotional features by the GCN efficiently. The capsule network can retain the exact spatial position information using the vector forms. Specifically, the CNN layer is replaced with the GCN layer, and the different depth features from the multiple GCN layers are used as the primary capsules. Moreover, to generate better classification capsules, a capsule attention mechanism is proposed to select the higher-quality primary capsules. The efficacy of the proposed GCN module and capsule attention module is validated by an ablation study in Section 3.2. Extensive experiments in Section 3.4 demonstrate that the proposed GCN-CA-CapsNet method achieves more satisfactory performance against the state-of-the-art methods and prove that the proposed method has an effective emotion feature learning capability from EEG–fNIRS.

It is well-known in the emotion literature that age, gender, and cultural differences among individuals affect the emotional response to a given stimulus [16,39]. In this study, 50 college students, including 25 males (age: 22.92 ± 1.71) and 25 females (age: 24.12 ± 1.67), volunteered and were recruited. They did not have significant differences in age, culture, or educational experience. However, the emotion recognition accuracy of the different subjects is obviously different in Figure 9. We furtherly study the correlation between different emotional responses and EEG/fNIRS brain signals for subjects with different genders and cultural differences and construct a transferable emotional brain signal feature learning model, so as to improve the accuracy and generalization of emotion recognition under cognitive differences.

## 5. Conclusions

In this paper, a novel EEG–fNIRS-based emotion recognition framework using a graph convolution and capsule attention network, namely GCN-CA-CapsNet, is introduced. Specifically, the CNN layer is replaced with the GCN layer, which can extract and fuse the graph structure features from the EEG and fNIRS. And the different depth features from the GCN layers are used as the primary capsules. Moreover, a capsule attention mechanism is proposed to give different primary capsules to different attention weights for feature fusion. So, the primary capsules with higher quality are selected to generate better classification capsules in the dynamic routing mechanism, and the recognition performance is improved. The efficacy of the proposed GCN module and capsule attention module is validated by an ablation study. Furthermore, the comparisons of the recognition results demonstrate that the proposed GCN-CA-CapsNet method achieves a more satisfactory performance against the state-of-the-art methods, and the best accuracy can reach 97.91%.

## Figures and Tables

**Table 2:** Comparison of ablation results.

Methods	Acc (%)					Number of Parameters	Times (s)
	Sad	Happy	Calm	Fear	Avg Acc (Std)		
CapsNet	91.27	92.71	90.34	92.42	91.69 (5.45)	2,897,155	1814
GCN-CapsNet	96.61	96.49	94.91	97.00	96.25 (3.04)	1,071,271	391
GCN-CA-CapsNet	97.76	98.19	97.06	98.62	97.91 (2.20)	1,102,585	429

**Table 3:** Performance comparison between EEG–fNIRS and single EEG/fNIRS.

Methods	Acc (%)				
	Sad	Happy	Calm	Fear	Avg Acc (Std)
GCN-CA-CapsNet (EEG)	96.31	96.47	95.03	96.88	96.17 (2.63)
GCN-CA-CapsNet (fNIRS)	83.10	88.19	81.41	85.95	84.66 (4.38)
GCN-CA-CapsNet (EEG-fNIRS)	97.76	98.19	97.06	98.62	97.91 (2.20)

**Table 4:** Performance comparison with the state-of-the-art methods.

Methods	Acc(%)				
	Sad	Happy	Calm	Fear	Avg Acc (Std)
GCN	90.15	90.71	88.45	91.33	90.16 (5.10)
TC-Net [13]	85.62	89.35	83.84	88.54	86.84 (9.25)
MLF-CapsNet [14]	94.48	94.79	93.46	95.86	94.65 (3.80)
ST-CapsNet [28]	93.57	95.02	93.04	94.42	94.01 (2.95)
GCN-CA-CapsNet	97.76	98.19	97.06	98.62	97.91 (2.20)

## Data Availability

The data presented in this study are available on request from the corresponding author due to privacy and ethical reasons. If you are interested in the dataset and want to use it, please download and fill out the license agreement (https://gitee.com/tycgj/enter) and send it to chenguijun@tyut.edu.cn. We will send you a download link through email after a review of your application.
